# Cytochrome P450 expression, induction and activity in human induced pluripotent stem cell-derived intestinal organoids and comparison with primary human intestinal epithelial cells and Caco-2 cells

**DOI:** 10.1007/s00204-020-02953-6

**Published:** 2020-12-02

**Authors:** Aafke W. F. Janssen, Loes P. M. Duivenvoorde, Deborah Rijkers, Rosalie Nijssen, Ad A. C. M. Peijnenburg, Meike van der Zande, Jochem Louisse

**Affiliations:** Wageningen Food Safety Research (WFSR), Wageningen University and Research, Akkermaalsbos 2, 6708 WB Wageningen, The Netherlands

**Keywords:** Cytochrome P450 (CYP), Gastrointestinal tract, Organotypic models, Stem cells, Toxicology

## Abstract

Human intestinal organoids (HIOs) are a promising in vitro model consisting of different intestinal cell types with a 3D microarchitecture resembling native tissue. In the current study, we aimed to assess the expression of the most common intestinal CYP enzymes in a human induced pluripotent stem cell (hiPSC)-derived HIO model, and the suitability of that model to study chemical-induced changes in CYP expression and activity. We compared this model with the commonly used human colonic adenocarcinoma cell line Caco-2 and with a human primary intestinal epithelial cell (IEC)-based model, closely resembling in vivo tissue. We optimized an existing protocol to differentiate hiPSCs into HIOs and demonstrated that obtained HIOs contain a polarized epithelium with tight junctions consisting of enterocytes, goblet cells, enteroendocrine cells and Paneth cells. We extensively characterized the gene expression of CYPs and activity of CYP3A4/5, indicating relatively high gene expression levels of the most important intestinal CYP enzymes in HIOs compared to the other models. Furthermore, we showed that *CYP1A1* and *CYP1B1* were induced by β-naphtoflavone in all three models, whereas *CYP3A4* was induced by phenobarbital and rifampicin in HIOs, in the IEC-based model (although not statistically significant), but not in Caco-2 cells. Interestingly, *CYP2B6* expression was not induced in any of the models by the well-known liver CYP2B6 inducer phenobarbital. In conclusion, our study indicates that hiPSC-based HIOs are a useful in vitro intestinal model to study biotransformation of chemicals in the intestine.

**Electronic supplementary material:**

The online version of this article (10.1007/s00204-020-02953-6) contains supplementary material, which is available to authorized users.

## Introduction

Cytochrome P450 (CYP) enzymes play crucial roles in detoxification or bioactivation of chemicals. Of the 18 CYP P450 families, members of the CYP 1–3 families are considered the main xenobiotic phase I metabolizing enzymes (Zanger and Schwab 2013). These enzymes are predominantly expressed in the liver, which is therefore the major organ for drug metabolism after oral exposure (Lin and Lu 2001; Paine et al. 2006). However, the intestine is regarded as the most important extrahepatic site of drug metabolism, especially upon oral exposure (Lin and Lu 2001; Paine et al. 2006). CYP enzymes are expressed in cells located at the tip of the villi and across the large surface area of the intestine, indicating that intestinal xenobiotic conversion can play an important role in overall first-pass metabolism (Murray et al. 1988; Xie et al. 2016).

In the small intestine, CYP3A4 has been observed to be the most abundant CYP enzyme. CYP2C9 and CYP3A5 have been reported to be the second most abundant enzymes, followed by CYP2C19, CYP2J2, CYP2D6 and CYP1A1 (Paine et al. 2006; Peters et al. 2016; Xie et al. 2016). However, large interindividual variations have been observed for, amongst others CYP3A5, CYP2D6 and CYP1A1, which were shown to be expressed in intestinal samples of some individuals, whereas they were absent in others (Paine et al. 2006; Peters et al. 2016; Xie et al. 2016). CYP expression and function is dependent on various factors, such as genetic polymorphisms, physiological factors (e.g., age, gender and (related) hormone levels) and pathological conditions (e.g., tumours and inflammation), which may impact chemical metabolism capacity (Cotreau et al. 2005; Zanger and Schwab 2013; Stavropoulou et al. 2018). Besides these factors, CYP functionality may also be affected by chemicals, e.g., by increasing their expression levels and/or inhibiting their activity, which may result in unanticipated adverse reactions and lower therapeutic efficacy of drugs (Lynch and Price 2007; Hansten 2018).

To date, human primary intestinal epithelial cells (IECs) are considered to be the best in vitro model to study intestinal metabolism (Grossmann et al. 2003), as well as primary IEC-derived models like the EpiIntestinal model (Ayehunie et al. 2018), as they represent the in vivo situation best. A disadvantage of human primary IECs is that they have been reported to survive for a limited time-frame, rapidly undergoing apoptosis when cultured ex vivo (Aldhous et al. 2001; Grossmann et al. 2003). This has been overcome by culturing the cells in a 3D-microtissue, like the human primary IEC-based EpiIntestinal model, which can be used for a longer time period. An alternative to the use of human primary cells is the use of cell lines, such as the human colonic adenocarcinoma cell line Caco-2, which is widely applied as a model to study intestinal transport, chemical metabolism and toxicity (Sun et al. 2008). Although Caco-2 cells form, upon differentiation, monolayers of polarized epithelial cells, which express various biotransformation enzymes, they generally show low biotransformation capacity and no (Prueksaritanont et al. 1996) or limited (Ozawa et al. 2015; Küblbeck et al. 2016) expression of CYP3A4, which is considered to play an important role in the intestinal metabolism of various chemicals, including midazolam and felodipine (Kato et al. 2003). This indicates that the Caco-2 model is limitedly suitable to study intestinal metabolism in vitro. Moreover, the Caco-2 model consists of a single cell type (enterocyte-like cells), and their typical culture in a 2D monolayer provides an in vitro intestinal tissue that lacks organ-specific microarchitecture and the physiological extracellular matrix environment (Eglen and Randle 2015).

Human intestinal organoids (HIOs) are promising novel in vitro models to study chemical metabolism, transport and toxicity. HIOs are 3D tissue structures with a microarchitecture and cellular composition resembling the native tissue (Spence et al. 2011; Finkbeiner et al. 2015; Tamminen et al. 2015). They can be generated by a stepwise differentiation process of human induced pluripotent stem cells (hiPSCs), by applying growth factors to mimic embryonic development (Sato et al. 2009, 2011; Mccracken et al. 2011; Finkbeiner et al. 2015). This differentiation process results in HIOs containing enterocytes, stem cells, goblet cells, Paneth cells, enteroendocrine cells and mesenchymal cells (Sato et al. 2009; Spence et al. 2011; Watson et al. 2014; Tamminen et al. 2015). The presence of mesenchymal cells enables crosstalk between epithelial and mesenchymal cells, which is known to have an essential role in in vivo intestinal development, for example in intestinal stem cell homeostasis (Kosinski et al. 2010; Le Guen et al. 2015; Meran et al. 2017). Therefore, hiPSC-derived HIOs are considered to be among the most promising in vitro models to assess chemical transport, metabolism and toxicity (Onozato et al. 2018). More specifically, these HIOs may overcome the limitations of the Caco-2 model regarding the limited expression of important biotransformation enzymes, such as CYP3A4.

In the current study we aimed to assess the suitability of hiPSC-derived HIOs as a model to study chemical-induced changes in CYP-expression and CYP activity, by characterizing the gene expression of the most common intestinal CYP enzymes and their inducibility in hiPSC-derived HIOs, as well as gene expression of some common intestinal enzymes involved in phase II metabolism. Caco-2 cells were used for comparison throughout all experiments and the EpiIntestinal model was used for comparison of the gene expression studies. HIOs were obtained based on differentiation methods described by McCracken et al. (2011), Spence et al. (2011) and Onozato et al. (2018), which were further optimized in the present study. HIOs were extensively characterized during the various differentiation stages and the optimisation of the differentiation method was supported by characterization of CYP and differentiation marker gene expression profiles and by immunohistochemical analysis. Using the optimized protocol, CYP expression was further evaluated in the absence or presence of typical CYP inducers (β-naphthoflavone, phenobarbital and rifampicin) and compared with expression in Caco-2 cells and the EpiIntestinal model. Induction of CYP3A4/5 activity was assessed by measuring midazolam hydroxylation (1′-OH-midazolam and 4-OH-midazolam formation) in HIOs and Caco-2 cells. The present study provides insight into whether HIOs are a relevant in vitro intestinal model regarding chemical metabolism and chemical-induced changes in expression and activity of biotransformation enzymes.

## Materials and methods

### Caco-2 cell culture

Human colorectal adenocarcinoma cells (Caco-2, HTB-37) were obtained from American Type Culture Collection (ATCC, Manassas, VA). Cells were cultured in a humidified incubator (37 °C, 5% CO_2_) in Dulbecco’s Modified Eagle’s Medium (DMEM) supplemented with 10% heat-inactivated fetal bovine serum (Gibco, Thermo Fisher Scientific, Waltham, MA), 1% non-essential amino acids (Gibco) and 1% penicillin/streptomycin (Sigma, St. Louis, MO). For experiments, Caco-2 cells were harvested at 80% confluence and seeded at a density of 4 × 10^5^ cells/cm^2^ into inserts with a polycarbonate membrane (0.4 µm pore, Corning, New York, NY). After 21 days of cultivation (using 500 µl in the apical and 1500 µl in the basolateral compartment), monolayer integrity was determined by measuring transepithelial electrical resistance (TEER) with a Millicell-ERS Volt-Ohm meter (Millipore). Only monolayers exhibiting TEER values exceeding 400 Ω cm^2^ were used for experiments.

### EpiIntestinal culture

The EpiIntestinal 3D tissue model of the human small intestine (MatTek Life Sciences, Ashland, USA) consists of enterocytes, Paneth cells, M cells, tuft cells and intestinal stem cells cultured in permeable cell culture inserts. Cell culture inserts (8.8 mm ID) were placed in 12-well plates and cells were maintained in 100 µl maintenance medium (MatTek Life Sciences) in the apical compartment and 5 ml maintenance medium in the basolateral compartment upon arrival. For CYP induction studies, maintenance medium was replaced with maintenance medium with CYP inducers 1 day after arrival.

### hiPSC culture

The hiPSC cell line (CS83iCTR-33n1) was provided by the Cedars-Sinai Medical Center’s David and Janet Polak Foundation Stem Cell Core Laboratory. These cells have been generated through episomal reprogramming of fibroblasts of a 31-year-old healthy female. The cell line has been fully characterized and no karyotype abnormalities have been found. hiPSCs were cultured on growth factor-reduced matrigel-coated (Corning) cell culture plates in mTeSR1 medium (Stem Cell Technologies, Vancouver, Canada) and were passaged using gentle cell dissociation reagent (Stem Cell Technologies).

### hiPSC differentiation into intestinal organoids

For differentiation, hiPSCs were dissociated into single cells using accutase (Stem Cell Technologies) and cultured on human embryonic stem cell qualified matrigel-coated 24-well plates in mTeSR1 supplemented with 10 µM Y-27632 (Stem Cell Technologies) for 1 day. hiPSCs were subsequently differentiated into definitive endoderm (DE) by incubation in RPMI1640 medium containing 1% non-essential amino acids (Gibco), 100 ng/ml Activin A (Cell Guidance Systems, Cambridge, UK) with increasing concentrations of fetal bovine serum (0%, 0.2% and 2% on day 1, 2 and 3, respectively). 15 ng/ml BMP4 (R&D Systems, Minneapolis, MN) was also added during the first day of definitive endoderm formation. Hindgut endoderm formation was induced by changing the medium to RPMI1640 medium containing 1% non-essential amino acids (Gibco), 2% fetal bovine serum, 500 ng/ml FGF4 (R&D Systems) and 3 µM Chiron99021 (Stemgent). After 4 days, free-floating spheroids were collected and embedded into domes of Matrigel (Corning). Spheroids were cultured in Advanced DMEM/F-12 containing 1 × B27 (Gibco), 1 × N2 (Gibco), 15 mM HEPES (Gibco), 1% penicillin/streptomycin (Gibco), 2 mM l-glutamine (Gibco), 50 ng/ml EGF (R&D Systems), 100 ng/ml Noggin (R&D Systems) and 500 ng/ml RSpondin-1 (R&D Systems). Medium was refreshed every 2–3 days and HIOs were passaged every 10–14 days. Organoids used for experiments where grown in Matrigel for at least 28 days unless otherwise stated. To promote intestinal differentiation, 0.5 µM A-83-01 (Sigma), 20 µM PD98059 (Stem Cell Technologies), 5 mM 5-aza-2′-deoxycytidine (Sigma), and 5 µM DAPT (Sigma) were added to the medium either from day 14 to 28 (according to Onozato et al. 2018), or after day 28 for a period of 3 or 7 days (further optimized protocol present study).

### RNA isolation and qPCR

Total RNA was extracted from the Caco-2 cells using the RNeasy Mini Kit (Qiagen, Venlo, The Netherlands). Total RNA was extracted from hiPSCs, organoids and EpiIntestinal tissues using the NucleoSpin RNA isolation kit (Macherey-Nagel, Düren, Germany). Subsequently, 200–500 ng RNA was used to synthesize cDNA using the iScript cDNA synthesis kit (Bio-Rad Laboratories, Veenendaal, The Netherlands). Changes in gene expression were determined by real-time PCR on a CFX384 real-time PCR detection system (Bio-Rad Laboratories) using SensiMix (Bioline; GC Biotech, Alphen aan den Rijn, The Netherlands). The PCR conditions consisted of an initial denaturation of 95 °C for 10 min, followed by 40 cycles of denaturation at 95 °C for 10 s and annealing extension at 60 °C for 15 s. The housekeeping gene RPL27 was used for normalization of gene expression when assessing the influence of time of differentiation on gene expression (HIOs), the gene expression of CYP enzymes when comparing culture protocols (HIOs) and the gene expression of CYP enzymes after chemical treatment (EpiIntestinal model, HIOs, Caco-2 cells). For comparison of gene expression of CYP and other selected phase I and phase II biotranformation enzymes between Caco-2 cells, HIOs and the EpiIntestinal model without chemical treatment, gene expression levels were normalized on Ct levels, since the expression of RPL27 was different in the different cell models. Primer sequences were taken from the Harvard PrimerBank and ordered from Eurogentec (Liège, Belgium). Sequences of the used primers are listed in Table 1.

**Table 1:** Primer sequences used for qPCR

Name	Primer sequence	
	Forward	Reverse
RPL27	ATCGCCAAGAGATCAAAGATAA	TCTGAAGACATCCTTATTGACG
OCT4	TTGGGCTCGAGAAGGATGTG	TCCTCTCGTTGTGCATAGTCG
NANOG	TTTGTGGGCCTGAAGAAAACT	AGGGCTGTCCTGAATAAGCAG
FOXA2	GGGTGATTGCTGGTCGTTT	ATACTGGAAGCCGAGTGCAT
SOX17	CCGAGTTGAGCAAGATGCTG	TGCATGTGCTGCACGCGCA
CXCR4	GGTGGTCTATGTTGGCGTCT	ACTGACGTTGGCAAAGATGA
GSC	CCTCCGCGAGGAGAAAGT	CGTTCTCCGACTCCTCTGAT
CDX2	CCAGCGGCGGAACCTGTG	GTCTTTCGTCCTGGTTTTCAC
KRT20	ACTAACGGAGCTGAGACGCA	GTAACGGGCCTTGGTCTCCT
KLF5	CATCCACTACTGCGATTACCC	CCCAGGTACACTTGTATGGC
IFABP2	CGCCCAAGGACAGACCTGAAT	TTCCAAGTGCTGTCAAACGCC
VIL1	CGGAAAGCACCCGTATGGAG	CGTCCACCACGCCTACATAG
SOX9	ATCAAGACGGAGCAGCTGAG	GGCTGTAGTGTGGGAGGTTG
LGR5	GGAAATCATGCCTTACAGAGC	CACTCCAAATGCACAGCACTG
LYZ	CCCTGGTCAGCCTAGCACTC	CCTTGCCCTGGACCGTAACA
MUC2	AGAAGGCACCGTATATGACGAC	CAGCGTTACAGACACACTGCTC
CHGA	TCCGACACACTTTCCAAGCC	TTCTGCTGATGTGCCCTCTC
FOXF1	CCCAGCATGTGTGACCGAAA	ATCACGCAAGGCTTGATGTCT
VIM	GGATTCACTCCCTCTGGTTG	TCGTGATGCTGAGAAGTTTCG
ACTA2	GTGTTGCCCCTGAAGAGCAT	GCTGGGACATTGAAAGTCTCA
CYP1A1	TCGGCCACGGAGTTTCTTC	GGTCAGCATGTGCCCAATCA
CYP1A2	ATGCTCAGCCTCGTGAAGAAC	GTTAGGCAGGTAGCGAAGGAT
CYP1B1	ACGTACCGGCCACTATCACT	CTCCCCACGACCTGATCCA
CYP2B6	CAGCCACCAGAACCTCAACC	AAGGTCGGAAAATCTCTGAATCTCATA
CYP2C9	CCTCTGGGGCATTATCCATC	ATATTTGCACAGTGAAACATAGGA
CYP2C19	ATTGAATGAAAACATCAGGATTG	GAGGGTTGTTGATGTCCATC
CYP2D6	CCTACGCTTCCAAAAGGCTTTT	AGAGAACAGGTCAGCCACCACT
CYP2J2	TGGCTTGCCCTTAATCAAAGAA	GGCCACTTGACATAATCAATCCA
CYP3A4	AAGTCGCCTCGAAGATACACA	AAGGAGAGAACACTGCTCGTG
CYP3A5	AATGTTTTGTCCTATCGTCAGGG	AGACCTTCGATTTGTGAAGACAG
AHR	CAAATCCTTCCAAGCGGCATA	CGCTGAGCCTAAGAACTGAAAG
CAR	GATGCTGGCATGAGGAAAGAC	TTGCTCCTTACTCAGTTGCAC
PXR	GCCCATGCTGAAATTCCACTA	GCCGATTGCATTCAATGTAGGA
CES1	ACCCCTGAGGTTTACTCCACC	TGCACATAGGAGGGTACGAGG
CES2	CTAGGTCCGCTGCGATTTG	TGAGGTCCTGTAGACACATGG
SULT1A1	GAGTTCAAAGCCCCAGGGATT	ACCTTGGCCATGTGGTAGAAG
SULT1A3	TGAGGTCAATGATCCAGGGGAA	CGCCTTTTCCATACGGTGGAAA
SULT1B1	CATCACCCCGGATTGTGAAAA	GGCATTACGAGCCAGATAAATCA
SULT1E1	GCCGGAATGCAAAGGATGTG	AGGAACCATAAGGAACCTGTCC
SULT2A1	CGTGATGAGTTCGTGATAAGGG	GGCAGAGAATCTCAGCCAACC
UGT1A1	TTGTCTGGCTGTTCCCACTTA	GGTCCGTCAGCATGACATCA
UGT1A10	GCCCCGTTCCTTTATGTGTGT	ATCTTCCAGAGTGTACGAGGTT
UGT1A7	CCTCCTTCCCCTATATGTGTGT	GCATCGGCAAAAACCATGAAC
UGT1A8	CAGCCCCATTCCCCTATGTGTTTC	GAGCATCGGCGAAATCCATGAAT
UGT1A9	TTCTCCAAACACCTGTTACGGAG	CCACAATTCCATGTTCTCCAG
UGT2B17	TTTATGAAAAGTTCGATAGATGGAC	CATCTTCACAGACTTTATATTATAGTCAG

### Immunofluorescence staining

Intestinal organoids were fixed in 4% paraformaldehyde (Electron Microscopy Sciences, Hatfield, PA), transferred to a 30% sucrose solution overnight at 4 °C, frozen in Tissue-Tek O.C.T. compound (Sakura Finetek Europe, Alphen aan den Rijn, the Netherlands) and cut in 10 µm sections. The sections were subsequently permeabilized in 0.5% Triton X-100 (Sigma) in Phosphate Buffered Saline (PBS, Gibco) and blocked using 5% normal donkey serum (Jackson ImmunoResearch, West Grove, PA) in 0.5% Triton X-100 in PBS. Primary antibodies were applied overnight at 4 °C in blocking buffer. The next day, sections were washed and incubated with appropriate secondary antibody for 1 h at room temperature. The antibodies including their dilutions are listed in Table 2. Nuclei were counterstained with 4′,6-diamidino-2-phenylindole (DAPI, Invitrogen). Finally, sections were washed and mounted in ProLong Diamond Antifade Mountant (Invitrogen). Confocal images were captured on a Zeiss LSM510 microscope.

**Table 2:** Antibodies used for immunofluorescence staining

Antibody	Manufacturer	Catalog number	Dilution
Goat anti-Ecadherin	R&D systems	AF648	1/500
Mouse anti-CDX2	BioGenex	MU392A-5UC	1/500
Rabbit anti-Chromogranin A	ImmunoStar	20085	1/500
Rabbit anti-ZO1	Invitrogen	40-2200	1/500
Mouse anti-Muc2	Santa Cruz	Sc515032	1/500
Mouse anti-Villin1	Abcam	Ab3304	1/25
Mouse anti-Vimentin	BD Pharmingen	550513	1/1000
Donkey anti-mouse Alexa Fluor 594	Invitrogen	A21203	1/1000
Donkey anti-rabbit Alexa Fluor 488	Invitrogen	A11055	1/1000
Donkey anti-goat Alexa Fluor 488	Invitrogen	A21206	1/1000

### Induction of CYP gene expression

For these studies, 24 h prior to exposure, three HIOs were pooled in one Matrigel dome. HIOs, the EpiIntestinal model and Caco-2 cells were exposed to 50 µM β-naphthoflavone (Sigma), 500 µM phenobarbital (Bipharma, Almere, the Netherlands) or 50 µM rifampicin (Supelco, Bellefonte, PA) for 48 h. Stock solutions were made in DMSO (Sigma) and final concentrations amounted to 0.1% in the medium. HIOs were exposed to 500 µl exposure medium. Caco-2 cells were exposed to 500 µl exposure medium in the apical and 1500 µl exposure medium in the basolateral compartment, and EpiIntestinal tissues were exposed to 50 µl exposure medium in the apical and 5 ml exposure medium in the basolateral compartment. Exposure medium was refreshed after 24 h. After incubation, HIOs, Caco-2 cells and the EpiIntestinal tissues were washed with PBS, collected and stored at − 80 °C before RNA isolation.

### Induction of CYP3A4/5 activity

CYP3A4/5 activity and its induction by rifampicin was determined by measuring the formation of 1′-hydroxymidazolam and 4-hydroxymidazolam in non-exposed and rifampicin-exposed HIOs and Caco-2 cells. For these studies, 24 h prior to exposure, three HIOs were pooled in one Matrigel dome. HIOs were exposed to 50 µM rifampicin for 48 h (final DMSO concentration: 0.1%). Subsequently, HIOs were removed from the Matrigel and each organoid was cut into four pieces. These organoids were transferred to 96-well ultra-low attachment microplates (Corning) and incubated in 200 µl Advanced DMEM/F-12 containing 1 × B27, 1 × N2, 15 mM HEPES, 1% penicillin/streptomycin, 2 mM l-glutamine, 50 ng/ml EGF (R&D Systems), 100 ng/ml Noggin (R&D Systems) and 500 ng/ml RSpondin-1 (R&D Systems) for 1 h. Subsequently, medium was replaced with fresh medium containing 50 µM midazolam (European Pharmacopoeia Reference, Strasbourg, France). After 24 h, 100 µl medium was collected and 100 µl ice-cold acetonitrile (Actu-All Chemicals, Oss, the Netherlands) was added to precipitate the proteins in the medium. The organoids were washed five times with PBS and organoid homogenates were prepared by lysing organoids in 250 µl ice-cold RIPA buffer (Pierce, Thermo Fisher Scientific). After centrifugation, 100 µl organoid lysate was collected and 100 µl acetonitrile was added to precipitate the proteins. The remainder of the cell lysate was used for protein quantification using the BCA Protein Assay kit (Pierce, Thermo Fisher Scientific) to normalize CYP3A/5 activity to protein levels.

After 21 days of differentiation, Caco-2 cells were exposed to 50 µM rifampicin for 48 h (final DMSO concentration: 0.1%). Culture medium was subsequently replaced with fresh DMEM containing 10% heat-inactivated fetal bovine serum, 1% non-essential amino acids, 1% penicillin/streptomycin and 50 µM midazolam (final DMSO concentration: 0.1%). After 24 h incubation, 100 µl medium was collected, (25 µl from the apical compartment and 75 µl from the basolateral compartments, reflecting the ratio of total volumes in the apical (500 µl) and basolateral (1500 µl) compartments), and 100 µl ice-cold acetonitrile was added to precipitate the proteins. The Caco-2 cells were washed five times with PBS and cell lysates were prepared by lysing Caco-2 cells in 250 µl ice-cold RIPA buffer (Pierce, Thermo Fisher Scientific). After centrifugation, 100 µl Caco-2 cell lysate was collected and 100 µl acetonitrile was added to precipitate the proteins. The remainder of the cell lysate was used for protein quantification using the BCA Protein Assay kit (Pierce, Thermo Fisher Scientific) to normalize CYP3A4/5 activity to protein levels.

### LC–MS analysis

The formation of 1′-OH-midazolam and 4-OH-midazolam was quantified in the culture medium and cell lysates of Caco-2 cells and HIOs using an Ultimate 3000 UHPLC system coupled to a Q-Exactive Orbitrap™-based mass spectrometer with a HESI-II electrospray operating in positive ion mode, using the vDIA method as described previously (Zomer and Mol 2015). The eluents for the LC separation were (A) water and (B) methanol:water 95:5 (v/v) both containing 2 mM ammonium formate and 20 µl formic acid per litre. The following gradient was used: 0% B until 0.1 min, then linear to 45% B in 1.9 min, followed by a rise to 100% B in 6 min. This condition was held for 6 min, after which a switch back to 0% B was performed in 0.5 min. After 4.5 min of equilibration, the next sample was injected. An Atlantis T3 LC column (3 µm particles, 100 × 3 mm) (Waters, Milford, MA, USA) was used. The LC flow rate was 300 µl/min, the LC column was kept at 40 °C and the injection volume was 5 µl. Under these conditions, retention times for 1′-OH-midazolam and 4-OH-midazolam were 10.1 and 9.8 min, respectively. Commercially available 1′-OH-midazolam (LGC Standards GmbH, Wesel, Germany) and 4-OH-midazolam (Sigma) were used to prepare standard curves. For quantification of 1′-OH-midazolam and 4-OH-midazolam, the protonated molecule (*m*/*z*: 342.08039) was used with a mass tolerance of ± 5 ppm. Additionally, fragments were used for identification of 1′-OH-midazolam (C_11_H_8_N_2_^+^ *m*/*z*: 168.06819) and 4-OH-midazolam (C_13_H_10_ClNF^+^ *m*/*z*: 234.0480). The software package Tracefinder (version 4.1, Thermo Scientific) was used to process the data.

### Statistical analysis

Data are presented as mean ± SEM. Comparisons between hiPSC-derived organoids and Caco-2 cells and between hiPSC-derived organoids with and without added small compounds were analysed using a two-tailed Student’s *t* test. A one-way ANOVA followed by Tukey’s post hoc multiple comparison test was used for comparisons between hiPSC-derived organoids, Caco-2 cells and the EpiIntestinal tissues and to determine statistically significant differences in the CYP induction experiments. *P* < 0.05 was considered as statistically significant. Prism software (version 5.02; GraphPad, San Diego, CA) was used for statistical analysis.

## Results

### Differentiation and characterization of intestinal organoids

**Fig. 1:**
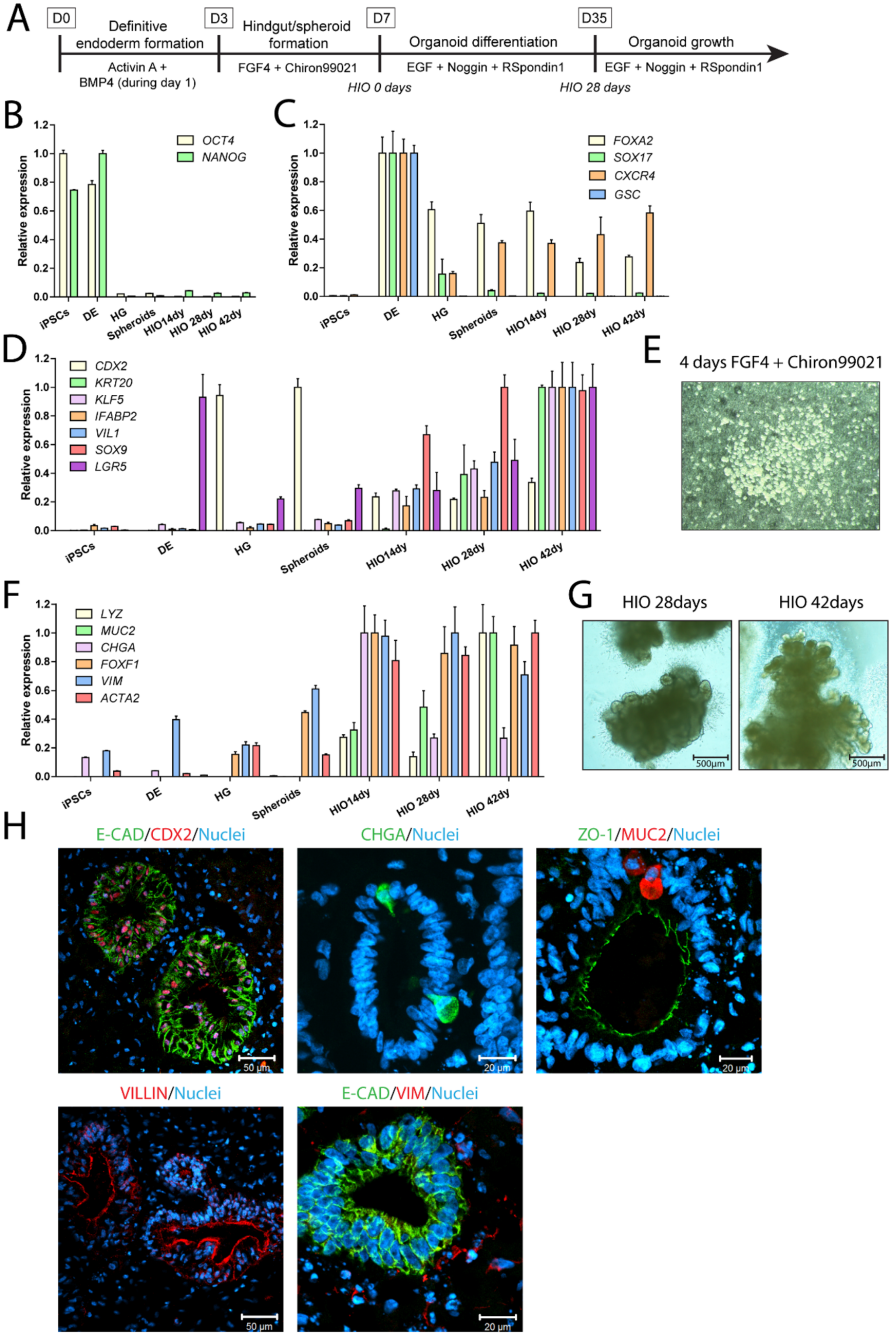
Differentiation of hiPSCs into human intestinal organoids (HIOs). **a** Schematic procedure for the differentiation of the hiPSC line CS83iCTR-33n1 into HIOs. Relative expression of **b** pluripotency and **c** definitive endoderm (DE) markers during the course of differentiation up to HIOs that were 42 days in 3D culture. The highest expression levels of each gene in each differentiation phase were set at one. HG, hindgut endoderm. **d** Relative expression of intestinal differentiation and crypt/stem cell markers during the course of differentiation up to HIOs that were 42 days in 3D culture. The highest expression levels of each gene in each differentiation phase were set at one. **e** Microscopic images of hiPSC-derived spheroids on day 7 of the differentiation procedure. **f** Relative expression of markers of Paneth cells (*LYZ*), goblet cells (*MUC2*), enteroendocrine cells (*CHGA*) and mesenchymal cells (*FOXF1*, *VIM* and *ACTA2*) during the course of differentiation up to HIOs that were 42 days in 3D culture. The highest expression levels of each gene in each differentiation phase were set at one. **g** Microscopic images of HIOs in 3D culture for 28 or 42 days. **h** Immunofluorescence stainings of various intestinal differentiation markers in HIOs that were in 3D culture for at least 28 days. Nuclei were counterstained with 4′,d-diamidino-2-phenylindole (DAPI). E-CAD: E-cadherin, CHGA: chromogranin A, ZO-1: zonula occludens-1, MUC2: mucin 2, VIM: vimentin. Data are mean values ± SEM from triplicate wells

The differentiation procedure of the human iPSC line CS83iCTR-33n1 into intestinal organoids was based on previous reports (Mccracken et al. 2011; Spence et al. 2011) (Fig. 1a). In the present study, the differentiation procedure was characterized by gene expression measurements and immunohistochemical evaluation. hiPSCs were first differentiated into definitive endoderm (DE) using the nodal-mimetic Activin A and BMP4. The provision of BMP4 to Activin A at the onset of differentiation promotes DE formation (Teo et al. 2012). Although mRNA levels of the pluripotency markers *OCT4* and *NANOG* were not altered in the generated DE cells (Fig. 1b), robust induction of the DE markers *FOXA2, SOX17, CXCR4* and *GSC* was observed (Fig. 1c). The DE cells were subsequently treated with FGF4 and Chiron99021 to induce hindgut endoderm formation and intestinal specification. Chiron99021 very potently inhibits the glycogen synthase kinase 3 (GSK3) pathway, resulting in the activation of WNT signalling and has been shown to be more potent in inducing hindgut endoderm formation than the more commonly used WNT3A (Tamminen et al. 2015). During the 4 days exposure to FGF4 and Chiron99021, the flat sheet of DE cells transformed into a hindgut endoderm culture, characterized by the expression of Caudal-related homeobox 2 (*CDX2*) (Fig. 1d), that started budding off and formed free-floating hindgut spheroids (Fig. 1e). Importantly, mRNA levels of pluripotency and definitive endoderm markers were lower in the hindgut endoderm culture and hindgut spheroids compared to the definitive endoderm stage (Fig. 1b, c). The spheroids were subsequently embedded into Matrigel (HIO 0 days) to promote intestinal differentiation and growth (Fig. 1g). After 14, 28 and 42 days in 3D culture, HIOs were harvested to determine gene expression levels of typical intestinal markers. As compared to spheroids, gene expression levels of intestinal differentiation markers *KRT20* (cellular protein specific for mature enterocytes and goblet cells), *KLF5* (transcription factor important for maintenance of barrier integrity and crypt architecture), *IFABP2* (cellular protein specific for mature enterocytes) and *VIL1* (major component brush border cytoskeleton specific for enterocytes) gradually increased over time in the developing HIOs (Fig. 1d). Although expression of the intestinal transcription factor *CDX2* was lower in HIOs in 3D culture than in spheroids (Fig. 1d), CDX2 was still clearly detected in virtually all epithelial cells (Fig. 1h). The intestinal crypt/stem cell markers *SOX9* and *LGR5* were both well expressed in the spheroids and expression gradually increased over time up to 42 days in HIO 3D culture. Interestingly, *LGR5* expression already peaked in the DE phase (Fig. 1d). In addition to enterocytes, the HIOs also contain Paneth cells, goblet cells and enteroendocrine cells as evidenced by expression of *LYZ, MUC2* and *CHGA,* respectively (Fig. 1f). Although *CHGA* expression peaked in HIOs that were 14 days in Matrigel, *CHGA* was still well expressed in HIOs that were 28 and 42 days in 3D culture, as evidenced by an average Ct value of 25 and by immunofluorescence staining showing the presence of CHGA + cells in the HIOs (Fig. 1f, h). Goblet cells (MUC2 +) were also identified microscopically in the epithelium of the HIOs (Fig. 1h). The presence of E-cadherin, ZO-1 and Villin1 located towards the apical surface of the enterocytes, demonstrates that the epithelial cells are polarized and formed tight junctions (Fig. 1h). Importantly, the increase in gene expression of *FOXF1, VIM* and *ACTA2* during HIO formation and the presence of Vimentin + cells indicated that the HIOs also contained mesenchymal cells (Fig. 1f, h). Although HIOs that were in 3D culture for 42 days obtained highest gene expression levels for the majority of the intestinal differentiation markers, genes related to the four major intestinal cell types (enterocytes, goblet cells, enteroendocrine and Paneth cells) were already well expressed after 28 days. Therefore, HIOs were kept at least 28 days in 3D culture before performing experiments, which is in line with McCracken et al. (2011) and Spence et al. (2011).

### CYP gene expression

**Fig. 2:**
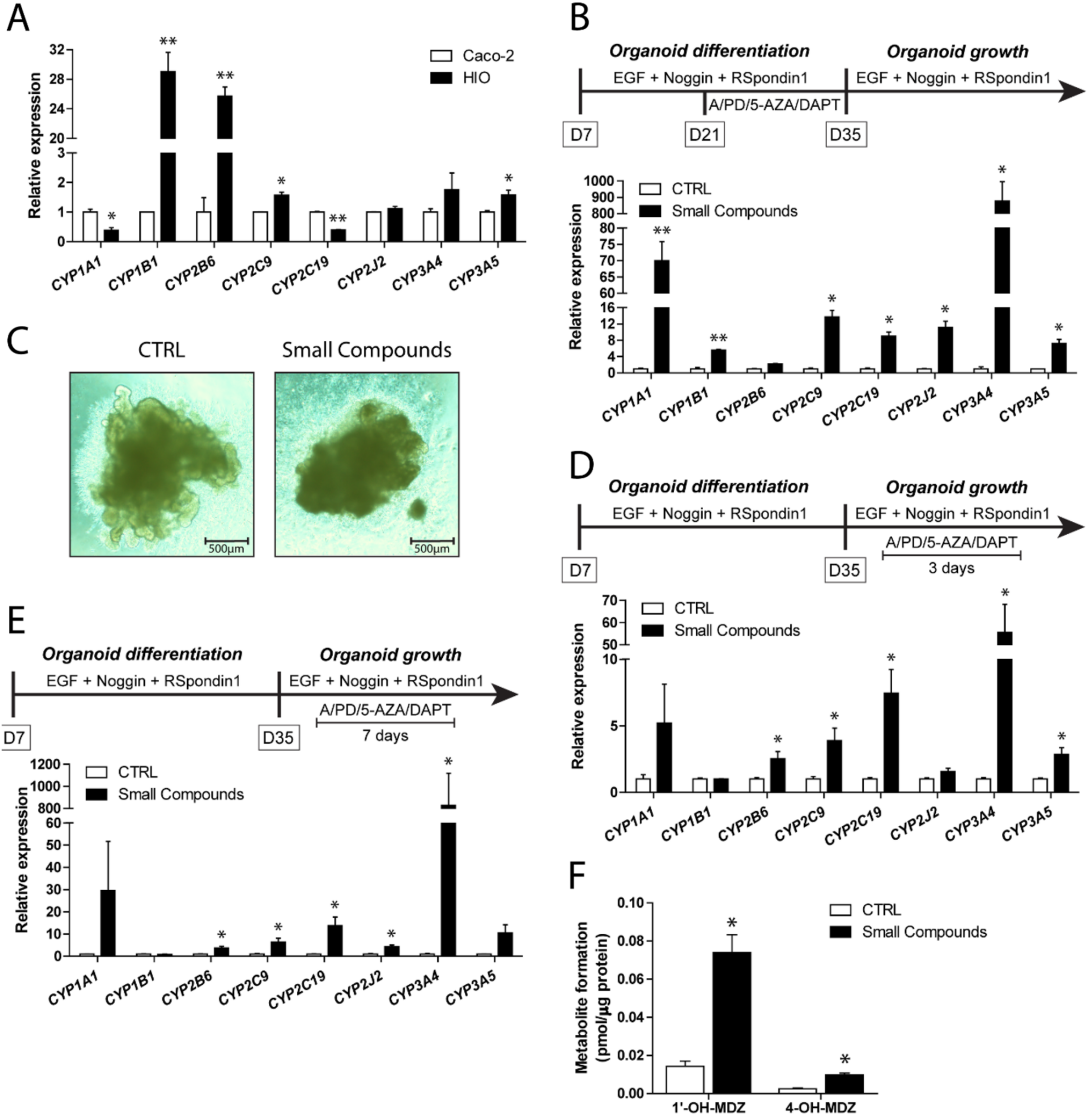
CYP gene expression in Caco-2 cells and HIOs. **a** Relative gene expression of common intestinal CYP enzymes in Caco-2 cells and HIOs. **b** Schematic procedure for HIO formation in the presence of the small-molecule compounds (A, A-83-01; PD, PD98059; 5-AZA, 5-aza-2′-deoxycytidine; DAPT) from day 21 to 35. Relative gene expression of common intestinal CYP enzymes in HIOs differentiated in the absence or presence of small-molecule compounds for 14 days. Gene expression levels in HIOs differentiated in the absence of small-molecule compounds (CTRL) were set at one. **c** Microscopic images of HIOs differentiated in the absence (CTRL) or presence of small-molecule compounds for 14 days. Schematic procedure for HIOs exposed to small-molecule compounds for **d** 3 or **e** 7 days after the organoid differentiation phase (after day 35) and relative gene expression of common intestinal CYP enzymes in HIOs exposed to small-molecule compounds. Gene expression levels in HIOs not exposed to small-molecule compounds (CTRL) were set at one. **f** CYP3A4/5 activity, quantified by 1′-OH-midazolam (1′-OH-MDZ) and 4-OH-midazolam (4-OH-MDZ) formation in HIO lysates and medium, in HIOs exposed to small-molecule compounds for 7 days. CYP3A4/5 activity in HIOs not exposed to small-molecule compounds (CTRL) are also presented. Asterisks indicate significantly different compared with Caco-2 cells or CTRL. *P < 0.05, **P < 0.001. Data are mean values ± SEM from triplicate wells

We determined the gene expression of a series of relevant intestinal CYP enzymes in HIOs and compared these with expression levels in Caco-2 cells. Whereas gene expression of *CYP1B1, CYP2B6, CYP2C9* and *CYP3A5* was significantly higher in HIOs, gene expression of *CYP1A1* and *CYP2C19* was significantly lower in the HIOs as compared to Caco-2 cells (Fig. 2a, Supplemental Table 1). Of note, both HIOs and Caco-2 cells did not express *CYP2D6* (data not shown), a gene containing the highest number of allele variants amongst the CYP enzymes resulting in *CYP2D6* gene deletion in approximately 5% of Asians, Africans and Caucasians (Ingelman-Sundberg et al. 2007). *CYP3A4*, with a Ct value of 32, showed limited expression in both Caco-2 cells and HIOs (Fig. 2a, Supplemental Table 1). Recently, Onozato et al. (2018) reported that small-molecule compounds (A-83-01, PD98059, 5-aza-2′-deoxycytidine and DAPT) induce expression of various genes in hiPSC-derived HIOs, including *CYP3A4*, when added to the medium during the final 15 days of the differentiation period [starting from HIOs of 12 days (differentiation day 19)]. Therefore, we also differentiated hiPSCs into HIOs in the presence or absence of small-molecule compounds and assessed the expression of the CYP genes. Adding small-molecule compounds during HIO differentiation from day 21 to 35 resulted in mRNA levels of *CYP3A4* that were about 900-fold higher as compared to HIOs differentiated in the absence of small-molecule compounds (Fig. 2b, Supplemental Table 2). In addition, small-molecule compounds robustly induced the expression of other CYP enzymes, including *CYP1A1, CYP1B1, CYP2C9, CYP2C19, CYP2J2* and *CYP3A5*, but not *CYP2B6* (Fig. 2b, Supplemental Table 2). A disadvantage of adding small-molecule compounds during HIO differentiation was that HIO growth almost completely stopped, indicating that the organoid culture cannot be further expanded and one would need to start differentiation from the hiPSCs if more organoids are needed. Also, the HIOs displayed a more compact morphology (Fig. 2c). Thus, we aimed to optimise the differentiation protocol into a protocol that can be used to keep a culture of well-growing HIOs, using the protocol as depicted in Fig. 1a, and supply fully maturated HIOs by treating a subset of the HIOs with small molecule compounds. To that end, HIOs were allowed to differentiate and grow for at least 35 days (28-day old HIOs) and were subsequently treated with small-molecule compounds for either 3 or 7 days. Figure 2d and e (Supplemental Tables 3 and 4) show that the relative mRNA levels in small molecule compounds-treated HIOs were similar for most CYPs when using a 7-day treatment with small-molecule compounds as when using the protocol of Onozato et al. (2018) (Fig. 2b, e), whereas these were lower when using a 3-day treatment with small-molecule compounds (Fig. 2d). For example, relative *CYP3A4* expression levels were about 60-fold higher than the control after 3 days incubation with small-molecule compounds, whereas 7 days incubation resulted in an 800-fold increase versus the control (Fig. 2d, e), being almost the same as the 900-fold increase versus the control in relative *CYP3A4* expression when using the protocol of Onozato et al. (2018) (Fig. 2b).

Interestingly, 7 days provision of small-molecule compounds also promoted HIO differentiation as displayed by elevated gene expression levels of intestinal differentiation markers, and markers for goblet cells and enteroendocrine cells (Supplemental Fig. 1a, b). These HIOs also contained a polarized epithelium with tight junctions and all different cell types (Supplemental Fig. 1c).

### Activity of CYP3A4/5 in HIOs treated with small-molecule compounds for 7 days

As CYP3A4 is considered to be one of the major CYP enzymes in intestinal metabolism, contributing to the clearance of various chemicals, we investigated whether provision of small-molecule compounds for 7 days also induced CYP3A4 (and CYP3A5) activity at the enzymatic level. To this end, HIOs were exposed for 24 h to the CYP3A4/5 substrate midazolam and subsequently the levels of 1′-OH-midazolam and 4-OH-midazolam were determined in HIO lysates and medium. 1′-OH-midazolam and 4-OH-midazolam levels were significantly higher after exposure to small-molecule compounds (Fig. 2f) indicating elevated CYP3A4/5 activity in these HIOs. Taken together, provision of small-molecule compounds for 7 days, after 28 days 3D culture, promotes differentiation of hiPSC-derived HIOs resulting in mature HIOs with CYP3A4/5 activity. Since this optimized protocol provides well-differentiated HIOs and more flexibility in culture, it was decided to continue the further studies using this optimized protocol (Fig. 2e).

### Gene expression in HIOs, Caco-2 cells and the EpiIntestinal model

**Fig. 3:**
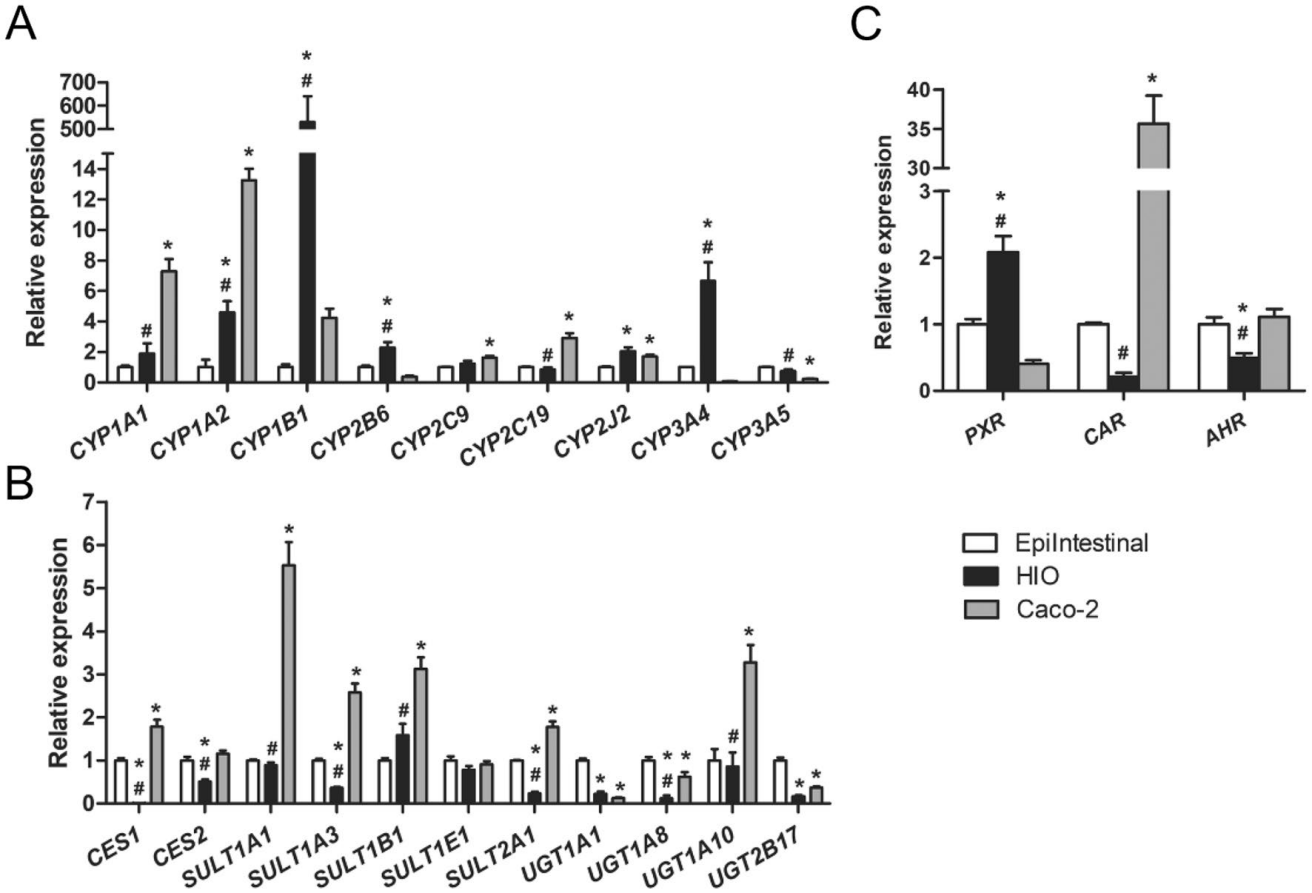
Gene expression in the EpiIntestinal model, HIOs and Caco-2 cells. **a** Relative expression of common intestinal CYP genes. **b** Relative expression of other selected biotransformation genes. **c** Relative expression of nuclear receptor genes. HIOs were exposed to small-molecule compounds for 7 days prior to mRNA extraction. Gene expression levels in the EpiIntestinal model were set at one. Asterisks indicate significant difference compared with the EpiIntestinal model (*P* < 0.05). Hashtags indicate significant difference between HIOs and Caco-2 cells (*P* < 0.05). Data are mean values ± SEM from triplicate wells

Gene expression of CYP genes and additionally some other genes coding for phase I biotransformation enzymes (carboxyl esterases (CEs)) and several common phase II biotransformation enzymes [uridine 5′-diphospho-glucuronosyltransferases (UGTs) and sulfotransferases (SULTs)] was compared between the HIOs cultured with the optimized protocol, the EpiIntestinal model, and Caco-2 cells. Comparison of the CYP genes showed significantly different expression of five out of nine genes between HIOs and the EpiIntestinal model, and of six out of nine genes between the Caco-2 cells and the EpiIntestinal model. The largest difference in CYP gene expression compared with the EpiIntestinal model was seen for *CYP1B1* (530 times higher in HIOs), *CYP1A2* (13 times higher in Caco-2 cells), *CYP1A1* (7 times higher in Caco-2 cells), and *CYP3A4* (7 times higher in HIOs) (Fig. 3a, Supplemental Table 5). When comparing the other genes for biotransformation enzymes, 7 out of 11 genes were differently expressed between HIOs and the EpiIntestinal model, and 9 out of 11 genes were differently expressed between Caco-2 cells and the EpiIntestinal model (Fig. 3b, Supplemental Table 5). Largest differences were observed for *CES1*, *UGT1A8*, *UGT2B17* and *UGT1A1* (125, 8, 6 and 4 times lower in HIOs, respectively) and for *SULT1A1*, *SULT1A3*, *SULT1B1* and *UGT1A10* (5, 3, 3 and 3 times higher in Caco-2 cells, respectively). Expression of the nuclear receptors aryl hydrocarbon receptor (AhR), constitutive androstane receptor (CAR, NR1I3) and pregnane X receptor (PXR, NR1I2), known to regulate the expression of various CYPs (Tompkins and Wallace 2007; Zanger and Schwab 2013), was also compared between the different models. Gene expression of *AHR* and *CAR* was lower in HIOs as compared to the EpiIntestinal model and the Caco-2 cells, whereas mRNA levels of *PXR* were higher in HIOs than in the EpiIntestinal model and the Caco-2 cells (Fig. 3c, Supplemental Table 5). In summary, HIOs cultured with small-molecule compounds for 7 days, after 28 days 3D culture, showed similar or higher mRNA levels of CYP enzymes, and similar or lower mRNA levels of a selection of other biotransformation enzymes compared to the EpiIntestinal model (Fig. 3, Supplemental Table 5).

### CYP induction in HIOs, Caco-2 cells and the EpiIntestinal model

**Fig. 4:**
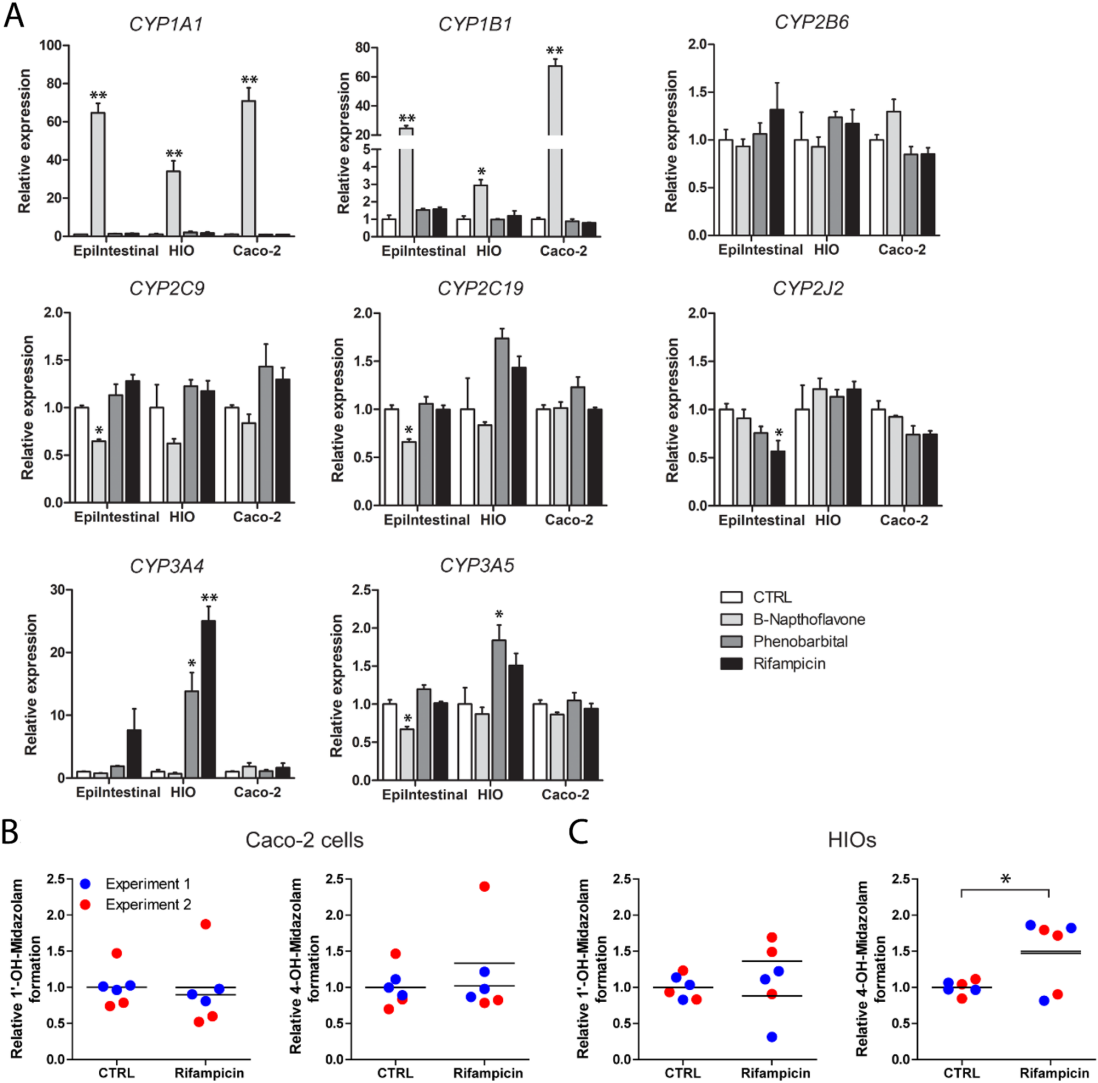
CYP induction in the EpiIntestinal model, HIOs and Caco-2 cells. **a** Relative expression of common intestinal CYP genes in the EpiIntestinal model, Caco-2 cells and HIOs treated with 50 µM β-naphthoflavone, 500 µM phenobarbital or 50 µM rifampicin for 48 h. HIOs were exposed to small-molecule compounds for 7 days prior to exposure to CYP inducers. Gene expression levels in the control samples were set at one. Data are presented as mean ± SEM from triplicates. Asterisks indicate significantly different compared with CTRL. *P < 0.05, **P < 0.001. **b** Caco-2 cells and **c** HIOs treated with 50 µM rifampicin for 48 h and subsequently with 50 µM midazolam for 24 h. HIOs were exposed to small-molecule compounds for 7 days prior to exposure to rifampicin and the entire experiment was performed twice (three replicates per experiment). CYP3A4/5 activity was quantified by 1′-OH-midazolam and 4-OH-midazolam formation in cell lysates and medium. All single data points and the means (lines) are presented. Asterisks indicate significantly different compared with the CTRL. *P < 0.05

To assess the inducibility of CYP enzymes by typical chemical CYP inducers in differentiated HIOs (HIOs exposed for 7 days to small-molecule compounds), Caco-2 cells and the EpiIntestinal model, all cell models were exposed to β-naphthoflavone, phenobarbital or rifampicin, which are typical inducers of CYP1A1/1A2/1B1, CYP2B6, and CYP3A4, respectively, in the human liver (Bernasconi et al. 2019). Relative gene expression levels compared to untreated controls (Supplemental Table 6) were determined and fold-changes were compared between the EpiIntestinal model, HIOs and Caco-2 cells (Fig. 4a). The typical CYP1 inducer β-naphthoflavone robustly induced expression of *CYP1A1* and *CYP1B1* in all models, but to the largest extent in Caco-2 cells (Fig. 4a). Interestingly, phenobarbital significantly induced *CYP3A4* in the HIOs, but had no effect on *CYP2B6* expression*.* No effect of phenobarbital was noticed in the Caco-2 cells or in the EpiIntestinal model (Fig. 4a). Rifampicin induced *CYP3A4* in the HIOs and in the EpiIntestinal model (although the induction was not significant in the latter), but not in Caco-2 cells (Fig. 4a). None of the CYP inducers was found to induce expression of *CYP2B6, CYP2C9, CYP2C19* or *CYP2J2* in all three models (Fig. 4a).

To assess whether the induction of *CYP3A4* gene expression levels by rifampicin also translated in elevated CYP3A4 activity, HIOs (and Caco-2 cells) were exposed to rifampicin and subsequently to the CYP3A4/5 substrate midazolam. In Caco-2 cells, rifampicin did not induce formation of 1′-OH-midazolam and 4-OH-midazolam (Fig. 4b). Surprisingly, in HIOs 1′-OH-midazolam levels were also not significantly altered upon rifampicin pre-treatment, but a significant increase in 4-OH-midazolam formation was observed (Fig. 4c).

## Discussion

In the present study, we improved an existing HIO differentiation protocol, leading towards better differentiation and expression of the most relevant intestinal CYP enzymes for xenobiotic metabolism without losing the capacity to keep them in prolonged culture, thus advancing throughput. Using this optimized protocol, we assessed the gene expression, induction and activity of important intestinal CYP enzymes in hiPSC-derived HIOs and compared these with the commonly used human colonic adenocarcinoma cell line Caco-2 and the human primary IEC-based EpiIntestinal model, closely resembling human small intestinal tissue (Ayehunie et al. 2018). The results of our study indicate that our HIO model expresses substantial levels of relevant intestinal CYP enzymes at the gene level, suggesting its relevance for in vitro biotransformation studies.

The results of the present study show that the gene expression of all evaluated CYPs, except *CYP2B6 * (and *CYP2D6*), increased upon treatment with the small-molecule compounds (A-83-01, PD98059, 5-aza-2′-deoxycytidine and DAPT), as reported before for *CYP3A4* by Onozato et al. (2018). Therefore, to study intestinal metabolism and/or chemical toxicity, pre-treatment of HIOs with small compounds is recommended. These small compounds have all been reported to promote intestinal differentiation (Ogaki et al. 2013; Iwao et al. 2015; Macedo et al. 2018). Indeed, relative expression of intestinal differentiation markers, including markers for enteroendocrine and goblet cells, was elevated upon small-molecule compounds treatment. A drawback of addition of the small compounds, using the method as described by Onozato et al. (2018), is that the expansion of the HIO culture largely reduces, indicating that the increased differentiation is directly correlated with a reduced cell proliferation. This means that the HIOs cannot be kept in culture and a new batch of HIOs has to be started for each experiment. This largely hampers the throughput of the method, so we aimed to improve the existing protocol towards a protocol that can keep proliferating HIOs in culture, using the protocol as depicted in Fig. 1a, from which a subset of matured HIOs can be derived in a rather short period to perform an experiment, by treatment with small molecule compounds. To that end, small-molecule compounds were not added during differentiation from day 21 to 35, as reported by Onozato et al. (2018), but instead HIOs were allowed to differentiate for at least 35 days (28 days in 3D culture) and subsequently a subset was treated with small-molecule compounds for 3 or 7 days. Using the protocol with exposure to small compounds for 7 days, expression of CYP genes was similar as when using the original protocol of Onozato et al. (2018). Furthermore, also differentiation of the HIOs appeared to be improved compared with the protocol without addition of small compounds. This 7-day treatment period was therefore selected as optimal protocol for further experiments.

In HIOs obtained with our protocol, *CYP3A4* gene expression was induced to a larger extent (25-fold) upon rifampicin treatment than reported for the HIO model by Onozato et al. (2018) (twofold). *CYP3A4* was hardly expressed in our Caco-2 cells and was also not induced by rifampicin, which is in line with other studies (Martin et al. 2008; Ozawa et al. 2015; Negoro et al. 2016). Limited expression of the pregnane X receptor (PXR), responsible for CYP3A4 induction by rifampicin (Goodwin et al. 1999), has been described to be responsible for the lack of CYP3A4 induction by rifampicin in Caco-2 cells (Pfrunder et al. 2003; Sun et al. 2008). Indeed, *PXR* was limitedly expressed in Caco-2 cells, as evidenced by an average Ct value of 31, whereas *PXR* gene expression levels were tenfold higher in our HIO model. In addition, in our study *CYP3A4* was also induced by phenobarbital in HIOs. Phenobarbital is a typical constitutive androstane receptor (CAR) agonist, resulting in CYP2B6 induction in the liver, which is used as a positive control in CYP induction studies in human hepatocytes (Bernasconi et al. 2019). The lack of *CYP2B6* induction by phenobarbital in our HIO model suggests that a functional CAR may be lacking. The induction of CYP3A4 by phenobarbital has been shown to be mediated also by PXR in human liver HepaRG cells (Li et al. 2019). Therefore, the phenobarbital-mediated induction of *CYP3A4* in HIOs, as measured in the present study, may be mediated by PXR as well.

To obtain insight whether *CYP3A4* expression in HIOs is relevant regarding expression in the in vivo situation, HIOs were compared to the EpiIntestinal model, shown to closely resemble human small intestine tissue explants, also with regard to *CYP3A4* mRNA expression (Ayehunie et al. 2018). Base level *CYP3A4* expression in the EpiIntestinal model was approximately seven times lower than in the HIOs. Exposure of the EpiIntestinal model to either rifampicin or phenobarbital led to a non-significant elevation in *CYP3A4* gene expression of approximately eight and two times, respectively. In the HIOs, a significant *CYP3A4* induction amounting to approximately 25 and 15 times was observed upon rifampicin and phenobarbital exposure, respectively. Based on these analyses, *CYP3A4* expression in the HIO model may be considered a bit high compared to the in vivo situation. However, the data should be interpreted with care due to complexity of comparing gene expression data from different models with also different expression levels of housekeeping genes. It must also be noted that interindividual differences in expression can be expected, and that the *CYP3A4* expression in the EpiIntestinal model or in the HIO model used in the present study may be from a donor with relatively low or high expression, respectively.

Transcriptional activation of *CYP1A* and *CYP1B* is mediated by the AhR. The induction of *CYP1A1* and *CYP1B1* gene expression by β-naphthoflavone was elevated in all three models, but was highest in Caco-2 cells. Although gene expression of *AHR* was slightly lower in the HIOs as compared to Caco-2 cells and the EpiIntestinal model, *CYP1B1* was, in comparison to the other two models, already highly expressed in HIOs prior to treatment with β-naphthoflavone, which may explain the relatively limited *CYP1B1* induction upon β-naphthoflavone treatment in HIOs.

We next evaluated if the induction in *CYP3A4* mRNA levels by rifampicin also translated into increased CYP3A4 activity. The conversion of midazolam into 1′-OH-midazolam is a commonly used method to determine CYP3A4/5 activity. In the literature, 1′-OH-midazolam hydroxylation of midazolam is often specifically linked to CYP3A4 activity, thereby neglecting CYP3A5 activity (Küblbeck et al. 2016; Yamaura et al. 2016; Jamwal et al. 2018; Onozato et al. 2018; Takayama et al. 2019). Albeit CYP3A4 is considered to be one of the most abundant intestinal CYP enzymes, the intestine also contains substantial CYP3A5 mRNA and protein levels (Canaparo et al. 2007; Peters et al. 2016). *CYP3A4* and *CYP3A5* mRNA levels were similar in our HIO model (exposed to small-molecule compounds for 7 days) (Supplemental Tables 4 and 5). Interestingly, although *CYP3A4* expression was limited in our Caco-2 cells, *CYP3A5* expression was substantially higher. To obtain more insight in relative CYP3A4 and CYP3A5 activities we determined the formation of both 1′-OH-midazolam and 4-OH-midazolam. Galetin et al. (2004) demonstrated, using CYP3A4 and CYP3A5 baculovirus-expressed recombinant systems, that the maximum velocity (*V*_max_) of 4-OH-midazolam formation was higher for CYP3A4 (2.5 pmol/min/pmol CYP3A4) than for CYP3A5 (0.5 pmol/min/pmol CYP3A5), and that the *V*_max_ of 1′-OH-midazolam formation was higher for CYP3A5 (6.7 pmol/min/pmol CYP3A5) than for CYP3A4 (2.0 pmol/min/pmol CYP3A4), indicating a preference of CYP3A4 for 4-OH-midazolam formation and a preference of CYP3A5 for 1′-OH-midazolam formation. Exposing HIOs to rifampicin for 48 h did not alter 1′-OH-midazolam formation upon subsequent incubation with midazolam, but significantly increased 4-OH-midazolam formation. Together with the elevated gene expression levels of *CYP3A4*, but not of *CYP3A5*, these data suggest that rifampicin induces CYP3A4 activity in HIOs resulting in increased conversion of midazolam into 4-OH-midazolam. In Caco-2 cells, however, rifampicin did not increase 1′-OH-midazolam nor 4-OH-midazolam levels.

Although the expression of *CYP3A4* mRNA and CYP3A4 activity are higher in HIOs than in Caco-2 cells, one cannot conclude that the HIO model outperforms the Caco-2 model in general. As shown in Fig. 3, gene expression of *CYP1B1*, *CES1*, *SULT2A1* and *UGT1A8* in Caco-2 cells resembled the gene expression in the EpiIntestinal model better than the expression in hiPSC-derived HIOs, suggesting that biotransformation of substrates of these enzymes may better be studied in Caco-2 cells than in the HIO model.

A striking difference in gene expression in the HIO model compared to the EpiIntestinal model was observed for *CYP1B1*, of which the expression was more than 500 times higher in the HIOs than in the EpiIntestinal model. CYP1B1 has been reported to play a role in the biotransformation of steroid hormones, fatty acids, vitamin A and melatonin (Li et al. 2017), and in the bioactivation of polycyclic aromatic hydrocarbons (Shimada et al. 1996). Although the present study does not indicate whether the high *CYP1B1* mRNA expression also translates to a high CYP1B1 activity, one should consider this characteristic of the model. In general, this indicates that extensive characterization of any model is of utmost importance to understand its strong and weak characteristics and its related applicability domain.

Since our HIOs were obtained from hiPSCs from one donor, it would be of interest for future studies to perform experiments in hiPSCs-derived HIOs from different donors, to obtain insight in inter-individual differences in biotransformation enzyme expression, induction and activity. Furthermore, it would also be of interest to compare the hiPSC-derived HIO model with HIOs derived from adult stem cells like the model described by Pleguezuelos-Manzano et al. (2020), to obtain more insight into which model most closely represents the in vivo situation.

Taken together, in the current study we generated an optimized protocol for hiPSC-derived HIOs containing substantial gene expression of relevant intestinal CYP enzymes, showing that *CYP1A1/1B1 g*ene expression can be induced by AhR activation and *CYP3A4* gene expression by PXR activation, whereas *CYP2B6* gene expression is not inducible, which may be due to a lack of functional CAR expression. Altogether, the data indicate that hiPSC-derived HIOs are useful models to study biotransformation of chemicals in the intestines.

## Electronic supplementary material

Below is the link to the electronic supplementary material.

Supplementary file1 (DOCX 1294 kb)

## Abbreviations

AhR: Aryl hydrocarbon receptor
CAR: Constitutive androstane receptor
CYP: Cytochrome P450
DE: Definitive endoderm
HG: Hindgut endoderm
IEC: Intestinal epithelial cells
hiPSCs: Human induced pluripotent stem cells
HIOs: Human intestinal organoids
PXR: Pregnane X receptor
