# Supplementary material for: Cytochrome P450 expression, induction and activity in human induced pluripotent stem cell-derived intestinal organoids and comparison with primary human intestinal epithelial cells and Caco-2 cells
Source: Arch Toxicol. 2020 Dec 2;95(3):907–22. doi: 10.1007/s00204-020-02953-6 (PMC7904554; doi:10.1007/s00204-020-02953-6)
Supplement: Supplementary file 1 — Supplementary file1 (DOCX 1294 kb) [file 204_2020_2953_MOESM1_ESM.docx]

**Supplementary data for:**

**Cytochrome P450 expression, induction and activity in human induced pluripotent stem cell-derived intestinal organoids and comparison with primary human intestinal epithelial cells and Caco-2 cells**

Aafke W.F. Janssen^1*^, Loes P.M. Duivenvoorde^1^, Deborah Rijkers^1^, Rosalie Nijssen^1^, Ad A.C.M. Peijnenburg^1^, Meike van der Zande^1#^, Jochem Louisse^1#^

Wageningen Food Safety Research (WFSR), Wageningen University and Research, Akkermaalsbos 2, 6708 WB Wageningen, The Netherlands

*** Corresponding author:**

Aafke Janssen PhD

Wageningen Food Safety Research

Akkermaalsbos 2

6708 WB Wageningen

The Netherlands

Phone: +31 317 483670

Email: aafke.janssen@wur.nl

# equal contribution

Supplemental table 1. Ct values of Cytochrome P450 enzymes in Caco-2 cells and human intestinal organoids.

| Gene | Ct value ± SEM |  |
| --- | --- | --- |
|  | Caco-2 | HIO |
| *CYP1A1* | 28.6 ± 0.16 | 30.2 ± 0.40 |
| *CYP1B1* | 34.6 ± 1.70 | 27.9 ± 0.13 |
| *CYP2B6* | 30.7 ± 0.91 | 25.6 ± 0.07 |
| *CYP2C9* | 28.2 ± 0.02 | 27.5 ± 0.10 |
| *CYP2C19* | 26.6 ± 0.03 | 28.0 ± 0.06 |
| *CYP2J2* | 29.3 ± 0.02 | 29.1 ± 0.10 |
| *CYP3A4* | 32.7 ± 0.16 | 32.1 ± 0.41 |
| *CYP3A5* | 29.4 ± 0.07 | 28.7 ± 0.17 |

Supplemental table 2. Ct values of Cytochrome P450 enzymes with and without induction with small compounds from day 21 to 35.

| Gene | Ct value CTRL ± SEM | Ct value Small Compounds ± SEM |
| --- | --- | --- |
| *CYP1A1* | 30.3 ± 0.27 | 25.1 ± 0.28 |
| *CYP1B1* | 26.9 ± 0.17 | 25.5 ± 0.17 |
| *CYP2B6* | 25.0 ± 0.15 | 25.1 ± 0.09 |
| *CYP2C9* | 26.7 ± 0.21 | 24.2 ± 0.10 |
| *CYP2C19* | 26.4 ± 0.22 | 24.4 ± 0.07 |
| *CYP2J2* | 29.7 ± 0.17 | 27.3 ± 0.13 |
| *CYP3A4* | 32.2 ± 0.76 | 23.2 ± 0.19 |
| *CYP3A5* | 29.3 ± 0.10 | 27.4 ± 0.08 |

Supplemental table 3. Ct values of Cytochrome P450 enzymes with and without induction with small compounds for 3 days.

| Gene | Ct value CTRL ± SEM | Ct value Small Compounds ± SEM |
| --- | --- | --- |
| *CYP1A1* | 33.7 ± 0.61 | 31.9 ± 0.63 |
| *CYP1B1* | 24.5 ± 0.15 | 24.8 ± 0.13 |
| *CYP2B6* | 27.6 ± 0.16 | 26.6 ± 0.26 |
| *CYP2C9* | 29.2 ± 0.26 | 27.5 ± 0.22 |
| *CYP2C19* | 30.0 ± 0.18 | 27.5 ± 0.27 |
| *CYP2J2* | 30.1 ± 0.12 | 29.8 ± 0.17 |
| *CYP3A4* | 35.3 ± 0.11 | 29.8 ± 0.48 |
| *CYP3A5* | 29.0 ± 0.09 | 27.9 ± 0.23 |

Supplemental table 4. Ct values of Cytochrome P450 enzymes with and without induction with small compounds for 1 week.

| Gene | Ct value CTRL ± SEM | Ct value Small Compounds ± SEM |
| --- | --- | --- |
| *CYP1A1* | 35.1 ± 0.06 | 31.1 ± 1.14 |
| *CYP1B1* | 22.5 ± 0.14 | 23.3 ± 0.43 |
| *CYP2B6* | 27.3 ± 0.19 | 25.2 ± 0.41 |
| *CYP2C9* | 27.9 ± 0.32 | 25.6 ± 0.50 |
| *CYP2C19* | 29.5 ± 0.09 | 26.3 ± 0.50 |
| *CYP2J2* | 29.2 ± 0.20 | 27.3 ± 0.35 |
| *CYP3A4* | 35.1 ± 0.38 | 25.9 ± 0.74 |
| *CYP3A5* | 28.8 ± 0.03 | 26.2 ± 0.72 |

Supplemental table 5. Ct values of housekeeping genes, phase I and II metabolism enzymes and nuclear receptors in the EpiIntestinal model, HIOs after induction with small compounds for 1 week and in Caco-2 cells after 21 days of differentiation.

| Gene | Ct value CTRL  EpiIntestinal ± SEM | Ct value CTRL  HIO ± SEM | Ct value CTRL Caco-2 ± SEM |
| --- | --- | --- | --- |
| *RPL27* | 18.7 ± 0.03 | 20.2 ± 0.06 | 18.1 ± 0.13 |
| *CYP1A1* | 32.6 ± 0.19 | 31.7 ± 0.66 | 29.3 ± 0.19 |
| *CYP1A2* | 35.2 ± 0.05 | 34.8 ± 0.25 | 33.5 ± 0.07 |
| *CYP1B1* | 34.1 ± 0.29 | 24.7 ± 0.32 | 31.9 ± 0.21 |
| *CYP2B6* | 28.3 ± 0.14 | 27.2 ± 0.24 | 29.6 ± 0.15 |
| *CYP2C9* | 26.6 ± 0.03 | 26.4 ± 0.26 | 25.9 ± 0.11 |
| *CYP2C19* | 26.6 ± 0.05 | 26.9 ± 0.26 | 25.0 ± 0.16 |
| *CYP2J2* | 29.4 ± 0.09 | 28.3 ± 0.19 | 28.6 ± 0.11 |
| *CYP3A4* | 29.2 ± 0.01 | 26.6 ± 0.30 | 32.9 ± 0.22 |
| *CYP3A5* | 26.4 ± 0.02 | 26.9 ± 0.29 | 28.6 ± 0.10 |
| *CES1* | 23.6 ± 0.09 | 30.8 ± 0.23 | 22.7 ± 0.14 |
| *CES2* | 26.8 ± 0.13 | 27.8 ± 0.13 | 26.6 ± 0.09 |
| *SULT1A1* | 27.6 ± 0.04 | 27.8 ± 0.11 | 25.0 ± 0.15 |
| *SULT1A3* | 22.4 ± 0.08 | 24.4 ± 0.14 | 20.6 ± 0.16 |
| *SULT1B1* | 27.9 ± 0.08 | 27.2 ± 0.28 | 26.2 ± 0.13 |
| *SULT1E1* | 27.1 ± 0.14 | 27.5 ± 0.19 | 27.2 ± 0.12 |
| *SULT2A1* | 29.3 ± 0.02 | 32.1 ± 0.31 | 28.2 ± 0.14 |
| *UGT1A1* | 27.8 ± 0.07 | 30.1 ± 0.46 | 30.9 ± 0.23 |
| *UGT1A8* | 33.1 ± 0.12 | 35.4 ± 0.11 | 33.8 ± 0.20 |
| *UGT1A10* | 35.4 ± 0.51 | 35.3 ± 0.43 | 33.5 ± 0.11 |
| *UGT2B17* | 26.2 ± 0.11 | 28.9 ± 0.22 | 27.7 ± 0.12 |
| *PXR* | 29.5 ± 0.11 | 28.4 ± 0.20 | 30.9 ± 0.22 |
| *CAR* | 31.0 ± 0.04 | 33.5 ± 0.51 | 25.6 ± 0.16 |
| *AHR* | 25.9 ± 0.15 | 26.9 ± 0.20 | 25.7 ± 0.16 |

Supplemental table 6. Ct values of Cytochrome P450 enzymes of controls in induction studies.

| Gene | Ct value CTRL EpiIntestinal ± SEM | Ct value CTRL  HIO ± SEM | Ct value CTRL Caco-2 ± SEM |
| --- | --- | --- | --- |
| *CYP1A1* | 32.3 ± 0.15 | 31.1 ± 0.25 | 29.6 ± 0.08 |
| *CYP1B1* | 34.3 ± 0.36 | 24.0 ± 0.26 | 30.0 ± 0.08 |
| *CYP2B6* | 28.6 ± 0.16 | 26.2 ± 0.48 | 30.4 ± 0.13 |
| *CYP2C9* | 27.1 ± 0.03 | 27.2 ± 0.40 | 30.2 ± 0.10 |
| *CYP2C19* | 27.6 ± 0.04 | 28.2 ± 0.55 | 28.6 ± 0.08 |
| *CYP2J2* | 29.6 ± 0.09 | 27.6 ± 0.37 | 29.2 ± 0.06 |
| *CYP3A4* | 29.6 ± 0.09 | 28.9 ± 0.50 | 35.5 ± 0.05 |
| *CYP3A5* | 26.6 ± 0.12 | 26.8 ± 0.33 | 24.7 ± 0.01 |

**
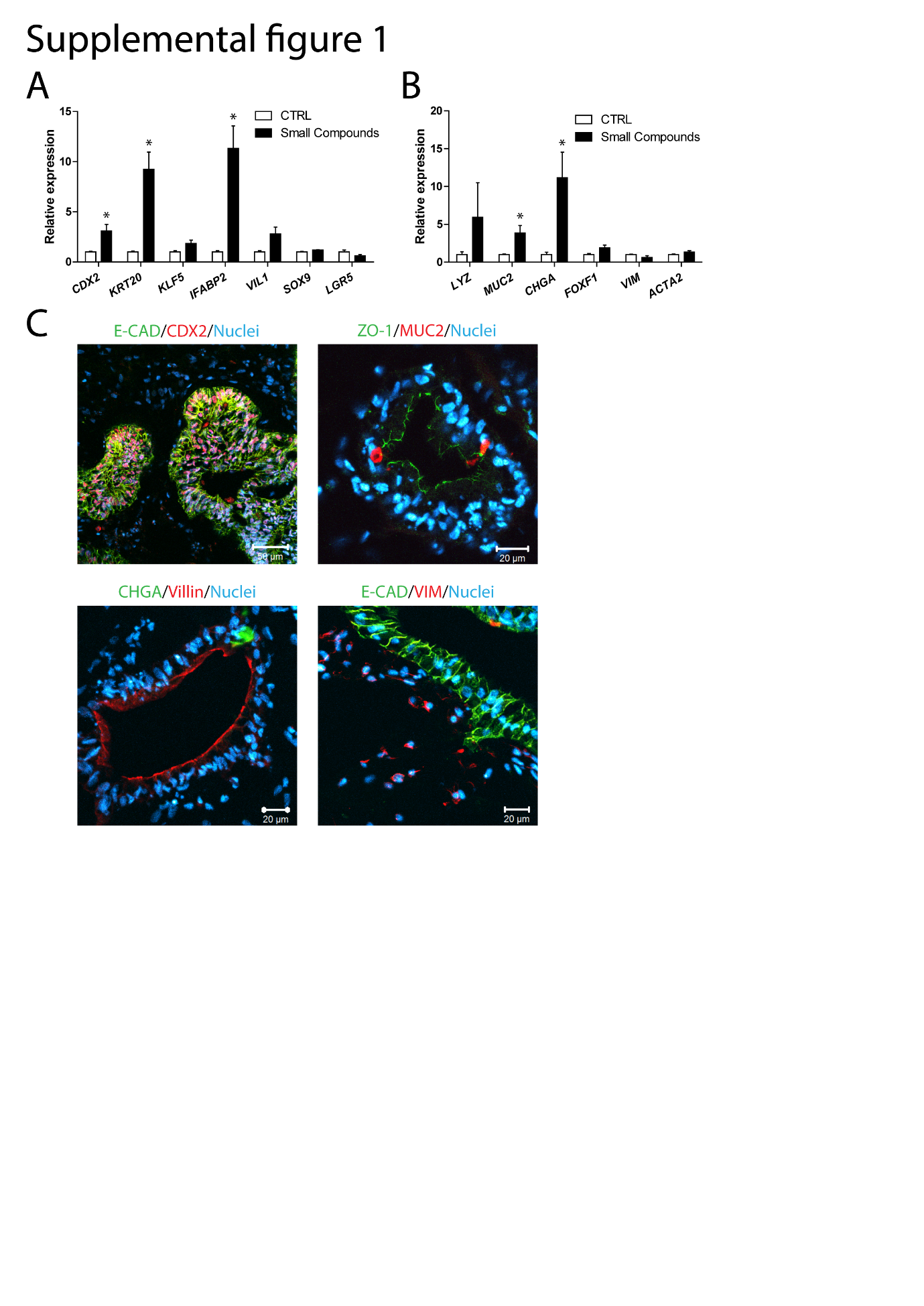
**

**Supplemental Figure 1. HIO characterization after 7 days exposure to small-molecule compounds.** Relative expression of genes related to **(A)** intestinal differentiation and crypt/stem cell markers and, **(B)** Paneth cells (*LYZ*), goblet cells (*MUC2*), enteroendocrine cells (*CHGA*) and mesenchymal cells (*FOXF1*, *VIM* and *ACTA2*) in the absence or presence of exposure to small-molecule compounds. Gene expression in HIOs not exposed to small-molecule compounds (CTRL) were set at one. Data are presented as mean ± SEM. Asterisks indicate significantly different compared with CTRL. *P<0.05, **P<0.001. **(C)** Immunofluorescence stainings of various intestinal differentiation markers in HIOs. Nuclei were counterstained with 4’,d-diamidino-2-phenylindole (DAPI). E-CAD, E-cadherin; ZO-1, zonula occludens-1; MUC2, mucin 2; CHGA, chromogranin A; VIM, vimentin. Data are mean values ± SEM from triplicate wells.
